# A branching process for homology distribution-based inference of polyploidy, speciation and loss

**DOI:** 10.1186/s13015-019-0153-8

**Published:** 2019-08-01

**Authors:** Yue Zhang, Chunfang Zheng, David Sankoff

**Affiliations:** Department of Mathematics and Statistics, University of Ottawa, 150 Louis Pasteur Pvt, Ottawa, K1N 6N5 Canada

**Keywords:** Branching process, Whole genome doubling, Fractionation, Solanaceae

## Abstract

**Background:**

The statistical distribution of the similarity or difference between pairs of paralogous genes, created by whole genome doubling, or between pairs of orthologous genes in two related species is an important source of information about genomic evolution, especially in plants.

**Methods:**

We derive the mixture of distributions of sequence similarity for duplicate gene pairs generated by repeated episodes of whole gene doubling. This involves integrating sequence divergence and gene pair loss through fractionation, using a branching process and a mutational model. We account not only for the timing of these events in terms of local modes, but also the amplitude and variance of the component distributions. This model is then extended to orthologous gene pairs.

**Results:**

We apply the model and inference procedures to the evolution of the Solanaceae, focusing on the genomes of economically important crops. We assess how consistent or variable fractionation rates are from species to species and over time.

## Background

An important source of information in the study of genomic evolution is the statistical distribution of the similarity or difference between pairs of *paralogous* genes, created by one or more rounds of polyploidization and resulting in whole genome doubling (WGD), tripling (WGT),…, or between pairs of *orthologous* genes, a consequence of speciation. In comparative genomics we try to identify peaks or local modes of these distributions, in order to assign chronological dates to each of the WGD or speciation events. Our approach has been to account for these data through processes of paralogous gene pair divergence by point mutation, and by gene pair loss through duplicate gene deletion—*fractionation*, in terms of a succession of multinomial samples integrated with a standard model of sequence divergence. This tries to account not only for the timing of peaks, but also their amplitude and how compact or diffuse they are [1–3]. In the present paper, we reformulate this model in terms of branching processes and extend it to the study of orthologous gene pairs, so that we can apply it to the evolution of the Solanaceae, focusing on the genomic comparisons among tomato, potato, eggplant, pepper, tobacco and petunia genomes. Our main goal is to systematically and quantitatively analyze the process of gene loss, using this family as an example, to assess how consistent or variable fractionation rates are from species to species and how they change over time.

We first review the classical discrete-time branching process and comment on how applicable it is for generating populations of paralogs. We also discuss the possibilities and limitations of statistical inference of the parameters of the model.

We then derive the expected counts of present-day paralogous pairs created at each ancestral time. These results are then reduced to simpler expressions (no summations, no factorials) for several important cases. We extend our model to introduce speciation, which allows us to derive the expected number of orthologous pairs with most recent common ancestors at each ancestral time.

In order to account for genomic data, we can observe all the paralogous pairs, as well as the orthologous pairs if two species are involved, but we cannot directly observe at which WGD or speciation time each pair originated. Here is where the mutational model plays a role. A paralog or ortholog pair does not consist of two identical genes, in terms of identical DNA sequence, but they are considerably more similar than two random sequences. However, the similarity decreases as the time from pair origination increases; nucleotide changes affect the DNA of both genes independently according to a relatively constant rate parameter. The set of pairs generated by a single WGD or speciation event displays a distribution of similarities, whose mean is inversely related to the time from that event to the present and whose variance reflects the degree of randomness of the process of similarity decay. The similarities of all the pairs originating from all the events thus constitute a mixture of distributions.

The means of the component distributions cannot usually be estimated by averaging, because of extensive overlap, but can be identified as local modes in the distribution of gene pair similarities. Maximum likelihood methods can then fill out the remaining information about the variances of each component distribution and their proportions in the mixture.

We apply our model and methodology to six genomes from the Solanaceae (“nightshade”) family of flowering plants using the grapevine genome as an outgroup. We compare all the genomes to each other (21 comparisons) and five of the six to themselves, using the SynMap tool on the CoGe platform [4, 5] to obtain the distribution of paralogous and orthologous gene pair similarities, resulting from WGD and speciation events. The goal is to estimate rates of fractionation, based on the information previously derived about the component distributions. We then compare the results from the 26 distributions for consistency and for variation between genomes.

## Methods

### The classical branching process in WGD context

In our process the discrete time parameter $i=1,2,\dots ,n$ is interpreted as the generation number and $X_i$ denotes the number of genes present in the *i*th generation. Generation $i+1$, for $1<i<n$ consists of the copies of genes in the $i{\rm th}$ generation as follows:

Each gene *j* in the *i*th generation produces a random number $\xi _j$ with distribution

1 $$\begin{aligned} u_k^{(i)}=P[\xi _j=k], \quad {\mathrm{for\ }} k=0,\ldots ,r_i, \end{aligned}$$

where $r_i \in \{2,3,\dots \}$ is the *ploidy* of the $i{\rm th}$ whole genome event. The distribution $u_{\cdot }^{(i)}$ depends on *i* and so may differ from generation to generation.

Let $X_1=1$, then for $i \ge 1$

2 $$\begin{aligned} X_{i+1}=\sum _{k=1}^{X_i} \xi _k. \end{aligned}$$

The mean and variance of the number of copies in the $i+1{\rm st}$ generation per gene in the *i*th generations are then

3 $$\begin{aligned} \mu _i=\sum _{k=0}^{r_i} ku_k^{(i)}, \quad {\sigma _i^2}=\sum _{k=0}^{r_i}(k-\mu _i)^2u_k^{(i)}. \end{aligned}$$

The generating function for this event, defined for $s\in [0,1]$, is

4 $$\begin{aligned} f(s)=E[s^{\xi }]=\sum _{k=0}^{r_i}u_k^{(i)}s^k, \end{aligned}$$

and so

5 $$\begin{aligned} f'(1)=\mu _i,\quad f''(1)={\sigma _2}-\mu _i,+\mu _i,^2 . \end{aligned}$$

Suppose $r_i$ and the $u_k$ are the same for every generation. The basic result on branching processes, dating from the 19th century work of Galton and Watson, is that the probability of eventual extinction is the smallest positive root of the fixed point equation

6 $$\begin{aligned} f(s)=s, \end{aligned}$$

which, in the biologically most relevant case $r_i\equiv 2$, becomes,

7 $$\begin{aligned} u_2s^2-(1-u_1)s +1-u_1-u_2=0, \end{aligned}$$

whose roots are

8 $$\begin{aligned} s = \frac{1-u_1 \pm \sqrt{(1-u_1)^2 -4u_2(1-u_1-u_2)}}{2u_2} \end{aligned}$$

9 $$= \left\{ 1, \frac{1-u_1}{u_2}-1\right\}.$$

This implies that the probability of extinction is less than 1 if and only if $u_0<u_2$.

In the other important case, $r_i\equiv 3$, we have

10 $$\begin{aligned} u_3s^3+u_2s^2-(1-u_1)s +1-u_1-u_2-u_3=0, \end{aligned}$$

where the solution is given by the pertinent cubic root.

### Applicability of a branching process model

A clear difference between classical branching processes and the WGD-fractionation process lies in the role of the time scale. Branching processes have a time scale made up of the positive integers, and all individuals in the population “reproduce” at the same time. WGD also affects all the genes in a genome synchronously, but it is a critical aspect for the analysis of fractionation that the $n-1$ WGD times are not limited to integers but may take on any real values between the starting time and the time of observation (or current time). We will circumvent this problem by considering the integer time scale of the branching process to represent the succession of generations in the population of genes, and by introducing a vector of event times, independent of the branching process. These event times will interact with the other model parameters during the inference procedures, but are not properly part of the model itself.

Another contrast between classical branching processes and the WGD-fractionation process, is that whereas the focus of branching process theory is the prediction of extinction over the long term, with our genome level studies we are generally interested in as few as one, but generally two, three or four events. And we are interested in fractionation in each generation and not the cumulative probability of eventual extinction.

Furthermore, our motivation is essentially an inference problem based on present-day genomes, but we have no access to gene families that have gone extinct; we cannot observe them in current genomes in order to analyze their genes.

Yet another difference is in the interpretation of the probabilities $u_k$. In the branching process model, these are the probabilities that any particular individual has *k* offspring. In WGD, on the other hand, all genes simultaneously give rise to exactly *r* copies, but the number that survive until the next event is governed by $u_k$. This reflects the fact that branching processes do not refer to anything between one branching event and the next, whereas after a WGD, fractionation takes place in the interval between that event and the next one. Despite this difference, on the formal level, there is no mathematical difference between the abstract model and the biological description.

Thus, though there are differences between branching processes and the biological phenomena of WGD and fractionation, the model fits the basic biology very well. A WGD occurs within an infinitesimal time period, a few generations, on the scale of evolutionary history spanning millions, tens of millions and hundreds of millions years, so the branching process with all individuals reproducing at the same time, is realistic. And the fractionation process, where many or most of the *r* duplicate genes are lost before the next WGD, can be nicely modelled by considering the $u_k$ to be survival probabilities rather than probabilities of offspring numbers.

Branching processes provide a realistic conceptual framework for the biological phenomena, but the biology in turn offers a novel kind of mathematical problem, namely to account for the ages of all the *pairs* of genes, i.e., the time they diverged from their latest common ancestor.

### The inference problem

This work is motivated by an interest in extracting information about evolutionary history from frequency distributions of homolog similarity scores. These distributions, depending on the particular type of score (similarity, $K_s$, 4DTv, $\dots$), are in fact mixtures of normals, or mixtures of some other kind of distribution, with non-negligible levels of noise, and subject to distortions and false signals of various types.

Dissecting mixtures of normals is a statistical problem that crops up in a number of fields, and there are standard techniques for carrying this out [6]. These methods, however, cannot necessarily adapt to field-specific constraints, not to mention noise inhomogeneous with respect to the similarity score, and other problems with the data. In comparative genomics, the distributions in the mixture tend to overlap to a large extent, the quality of the data diminishes and noise level increases with lower similarity score. The variance of the component distributions increases with lower similarity score, but not in an entirely predictable manner.

Nevertheless, as we shall try to demonstrate, it is feasible to pin down the dates of at least four WGD and speciation events in the history of a genome. For each of the mixture components originating with these events we can estimate a mean, a variance, and a proportion, the latter referring to the area under the component distribution as a proportion of the total area. Only the mean and the proportion turn out to be useful statistics in our eventual analysis of fractionation, meaning that we can only estimate two parameters in the model for each component in the mixture of distributions. In addition, another data item is sometimes available, the current number of unpaired genes, which should allow the estimation of an additional parameter affecting the most recent WGD or speciation event [2]. The biologically motivated constraint $u_0=0$ can be invoked to allow estimation of $u_2$, even though this “no lineage extinction” condition is an overstatement, given that not all genes are essential, and that occasionally both copies of a gene are lost.

### Details of the branching process—the evolution of population size

Denote by $M_1,\dots ,M_n$ the total number of individuals (genes) existing in the population at generation $1, 2\dots ,n$.

To get from generation *i* to generation $i+1$, for $i=1,\dots ,n-1$, each of the population of $M_i$ genes is first replaced by $r_i\ge 2$ progeny. We call $r_i$ the *ploidy* of the event. We denote by $u_j^{(i)}$ the probability that *j* of these $r_i$ progeny survive until generation $i+1$, for $j=0,\dots ,r_i$. (In applying this model we often assume $u_0^{(i)}=0$—“no lineage extinction”—so that we gain a degree of freedom for estimating other parameters. But this constraint is not really required in the model.) There is no replacement event at the *nth* and final generation; this is simply the point at which the population is observed.

Let $a_0^{(i)},\dots , a_{r_i}^{(i)}$ be the number of genes at generation *i*, of which $0,\dots ,r_i$, respectively, survive until generation $i+1$, so that

11 $$\begin{aligned} M_i=\sum _{j=0}^{r_i}{a_j^{(i)}},\ \ \ \ \ M_{i+1}=\sum _{j=0}^{r_i}j{a_j^{(i)}}. \end{aligned}$$

The probability distribution of the evolutionary histories represented by ${\mathbf {r}}=\{r_i\}_{i=1\dots n-1}$ and the variable ${\mathbf {a}}=\{a_j^{(i)}\}_{j=0\dots r_i}^{i=1\dots n-1}$ is

12 $$\begin{aligned} P(\mathbf {r;a})= \prod _{i=1}^{n-1}\Bigg [\left( {\begin{array}{c}M_i\\ a_0^{(i)},\dots ,a_{r_i}^{(i)}\end{array}}\right) \prod _{j=1}^{r_i} (u_j^{(i)})^{a_j^{(i)}}\Bigg ], \end{aligned}$$

as can be proved by induction on *i*. The expected number of genes at generation *n* is

13 $$\begin{aligned} {\mathbf {E}}(M_n)=\sum _{{\mathbf {a}}}P(\mathbf {r;a})M_n . \end{aligned}$$

Similarly, for the events starting at generation *j* with $M_j$ genes, up to generation *k*, we write

14 $$\begin{aligned} P^{(j,k)}(\mathbf {r;a})= & {} \prod _{i=j}^{k-1}\Bigg [\left( {\begin{array}{c}M_i\\ a_0^{(i)},\dots ,a_{r_i}^{(i)}\end{array}}\right) \prod _{h=1}^{r_i} (u_h^{(i)})^{a_h^{(i)}}\Bigg ] \nonumber \\ {\mathbf {E}}^{(j,k)}(M_k)= & {} \sum _{{\mathbf {a}}}P^{(j,k)}(\mathbf {r;a})m_k. \end{aligned}$$

### Paralogous gene pairs

Having described the origin and survival of individual genes, we now summarize the analysis in [2] of the *pairs* of genes observed at generation *n* whose most recent common ancestor was replaced by $r_i$ progeny at some generation *i*.

For each of the $a_j^{(i)}$ genes with $j\ge 2$ surviving copies, there are $$\left( {\begin{array}{c}j\\ 2\end{array}}\right)$$ surviving pairs of genes at generation $i+1$. The total number of pairs created at generation *i* and surviving to generation $i+1$ is thus

15 $$\begin{aligned} d^{(i,i+1)}=\sum _{j=2}^{r_i}\left( {\begin{array}{c}j\\ 2\end{array}}\right) a_j^{(i)}. \end{aligned}$$

These are called the *i*-pairs at generation $i+1$. The expected number of such pairs is

16 $$\begin{aligned} {{\mathbf {E}}}(d^{(i,i+1)})=\sum _{{\mathbf {a}}}P^{(1,i+1)}(\mathbf {r;a}) \sum _{j=2}^{r_i}\left( {\begin{array}{c}j\\ 2\end{array}}\right) a_j^{(i)}. \end{aligned}$$

At generation *j*, for $i+1\le j\le n$, any two descendants of the two genes making up a *i*-pair *with no more recent common ancestor* is also called a *i*-pair (at generation *j*). In other words, for any two genes at generation *j*, they form an *i*-pair if their most recent common ancestor underwent replacement at generation *i*.

For a given *i*-pair $g'$ and $g''$ at generation $i+1$, where $i<n-1$, the expected number of pairs of descendants $d^{(i,n)}$ having no more recent common ancestor is

17 $$\begin{aligned} {{\mathbf {E}}}(d^{(i,n)})={{\mathbf {E}}}(d^{(i,i+1)})\big ({\mathbf {E}}^{(i+1,n)}(M_n)\big )^2 \end{aligned}$$

where $M_{i+1}=1$ in both factors representing the descendants of an *i*-pair. This follows from the independence among the fractionation process between generation *i* and $i+1$ and both processes starting with $g'$ and $g''$.

Of the $M_n$ genes in Eq. (13), the expected number of unpaired genes is

18 $$\begin{aligned} {{\mathbf {E}}}(M^*)=M_1 \prod _{i=1}^{n-1}u_1^{(i)}. \end{aligned}$$

### Reductions to simple form

The accumulation of multinomial coefficients in Eq. (12), and the potentially high degree polynomials might seem computationally formidable. In practice, however, *n* seldom attains 5 or 6, and the $r_i$ are generally 2 or 3. Thus individual instances of the model are generally computationally tractable. In addition, though Eq. (17) would seem to entail an increasing complexity of formulae as *n* increases, in many important cases this reduces to simple expressions.

*Successive doublings (Tetraploidizations)* For example if all $r_i=2$ for $1\le i \le n-1$, we have by induction that Eq. (17) reduces to

#### **Theorem 1**

19 $$\begin{aligned} {\mathrm{E}}(N_1) & = u_2^{(1)}{\mathrm{\Pi }}_{j=2}^{n-1}(1+u_2^{(j)} )^2\nonumber \\ {\mathrm{E}}(N_i) & = {\mathrm{\Pi }}_{j=1}^{i-1}(1+u_2^{(j)})u_2^{(i)}\mathrm{\Pi }_{j=i+1}^{n-1}(1+u_2^{(j)} )^2 \nonumber \\ {\mathrm{E}}(N_{n-1}) & = u_2^{(n-1)}\mathrm{\Pi }_{j=1}^{n-2}(1+u_2^{(j)}), \end{aligned}$$

*where* $N_i$ *is the expected number of duplicate pairs of genes produced at generation* *i* *surviving until generation* *n*.

#### **Corollary 1**

*If all the* $u_2^{(j)}= u$, *then for* $1\le i\le n-1$,

20 $$\begin{aligned} {\mathrm{E}}(N_i )=u(1+u)^{2n-i-1}. \end{aligned}$$

*Successive triplings (Hexaploidizations)* In the case all $r_i=3$ for $1\le i \le n-1$,

21 $$\begin{aligned} {\mathrm{E}}(N_1) & =(3u_3^{(1)}+u_2^{(1)})\mathrm{\Pi }_{j=2}^{n-1}\nonumber \\& \quad (1+2u_3^{(j)}+u_2^{(j)})^2\nonumber \\ {\mathrm{E}}(N_i)&=\mathrm{\Pi }_{j=1}^{i-1}(1+2u_3^{(j)}+u_2^{(j)})\nonumber \\& \quad (3u_3^{(i)}+u_2^{(i)})\mathrm{\Pi }_{j=i+1}^{n-1}(1+2u_3^{(j)}+u_2^{(j)})^2 \nonumber \\ {\mathrm{E}}(N_{n-1}) &=(3u_3^{(n-1)}+u_2^{(n-1)})\mathrm{\Pi }_{j=1}^{n-2}\nonumber \\& \quad (1+2u_3^{(j)}+u_2^{(j)}). \end{aligned}$$

*General* *r*. For $r\ge 2$ the same for all generations, and $u_j^{(i)}=u_j$ for $j=1,\dots ,r$ and $i=1,\dots n-1$, there will be coefficients $K\ge 0,$ the expected number of gene pairs between  *t*_*i* − 1_ and *t*_*i*_, and $K'\ge 0$, the expected number of genes between *t*_*j*_ and *t*_*j* + 1_, depending on the distribution of $u_j$, such that

22 $$\begin{aligned} {\mathrm{E}}(N_i)=K'{K}^{2n-i-1}. \end{aligned}$$

### Introducing speciation into the model

**Fig. 1:**
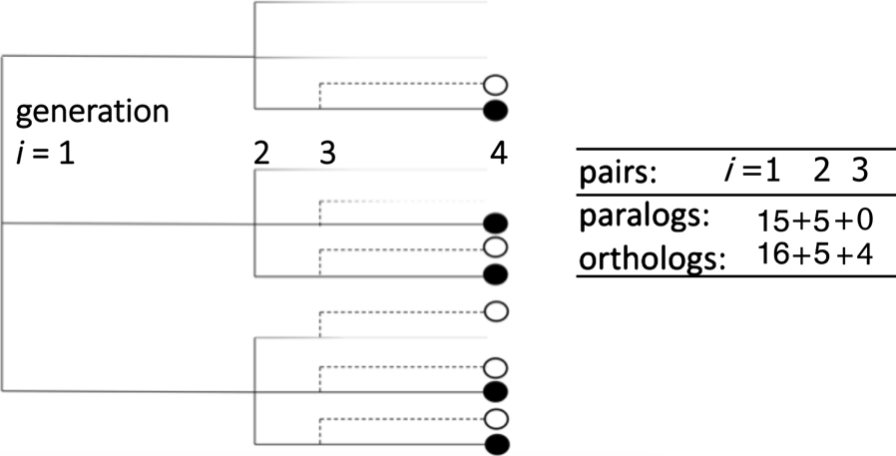
A gene tree produced by two triplings at generations 1 and 2, followed by a speciation at generation 3, showing the number of paralogous and orthologous 1-, 2- and 3-pairs. The generation of origin of any paralogous pair (same colour dots) or orthologous pair (different colour dots) is that of its most recent common ancestor

When two populations of a species evolve into two daughter species, we may assume that they initially have the same gene complement, and share identical paralog trees. We can no longer observe the state of the paralog tree at generation *n*—that event is in the past—instead we observe the current set of orthologous gene pairs at generation $n+1$. Obviously, if such a tree has $M_n$ genes at generation *n*, this will create at most $M_n$ different orthologous *n*-pairs at generation $n+1$, the time of observation, taking into account the possibility of fractionation between the *n*-th and $n+1$-st generations. Thus in Fig. 1, though there are six genes in generation 3, we only observe four pairs of orthologs surviving fractionation after the speciation event.

One way to allow fractionation to continue beyond the speciation event is to extend the branching process, treating speciation as another WGD event, though the counting of orthologs is necessarily different than the counting of *i*-paralogs, as illustrated in Fig. 1.

For this sequence of events, the same logic behind Eqs. (19–22) allows us to write

23 $$\begin{aligned} \mathrm{E}(O_1) & = 0.5(3u_3^{(1)}+u_2^{(1)})(1+2u_3^{(2)}+u_2^{(2)})^2(1+u_2^{(3)})^2\nonumber \\ {\mathrm{E}}(O_2) & = 0.5(1+2u^{(1)}_3+u_2^{(1)})(3u_3^{(2)}+u_2^{(2)}) (1+u_2^{(3)})^2\nonumber \\ \mathrm{E}(O_3) & = u_2^{(3)}(1+2u_3^{(1)}+u_2^{(1)})(1+2u^{(2)}_3+u_2^{(2)}), \end{aligned}$$

where *O* stands for the number of ortholog pairs.

This approach is more general than simply counting two pairs of orthologs for every pair of paralogs required by the no fractionation assumption, since $u_2^{(3)}$ can be less than 1. However, even this is not really satisfactory, since it treats gene loss in one of the two genomes created at generation *n* as if it were the product of fractionation within a single genome, when in fact the two genomes are entirely independent of each other. The “correct” way of proceeding would be to allow the fractionation regime operative between the $n-1$-st and *n*-th generations to continue independently between the *n*-th and $n+1$-st generations in each of the two genomes until the observation step $n+1$, or until this is interrupted by new WGDs in the two species. This is done as follows

For example, suppose there is just $M_1=1$ gene at generation 1, and suppose all $r_i=2$. We can write $u(i)=u_2(i), i=1,\dots , n-1$ for the probability that both progeny of a gene at generation *i* survive until generation $i+1$. We rewrite Eq. (19) as

24 $$\begin{aligned}&{{\mathbf {E}}}(N_1) =u(1){\mathrm{\Pi }}_{j=2}^{n-1}(1+u(j) )^2\ \ \nonumber \\&{{\mathbf {E}}}(N_i)={\mathrm{\Pi }}_{j=1}^{i-1}(1+u(j))u(i)\mathrm{\Pi }_{j=i+1}^{n-1}(1+u(j) )^2\ \ \nonumber \\&{{\mathbf {E}}}(N_{n-1}) =\mathrm{\Pi }_{j=1}^{n-2}(1+u(j))u(n-1) \end{aligned}$$

Set $s=n$ to emphasize that this is a speciation event, and not a WGD or observation event. Suppose there are $n_A-1-s$ WGD in species *A* after speciation and $n_B-1-s$ in species *B*. Let

25 $$\begin{aligned} F_A & = \Pi _{i=s}^{n_A-1}(1+u^A(i))\nonumber \\ F_B & = \Pi _{k=s}^{n_B-1}(1+u^B(k)) \end{aligned}$$

be the expectation of the “amplifying factors” affecting the distribution of orthologs due to these WGD. Then

26 $$\begin{aligned} {{\mathbf {E}}}(O_1 )&= \frac{1}{2}u(1)\Pi _{j=2}^{s-1}(1+u(j) )^2F_AF_B \nonumber \\ {{\mathbf {E}}}(O_i)&=\frac{1}{2}\Pi _{j=1}^{i-1}(1+u(j))u(i) \Pi _{j=i+1}^{s-1}\nonumber \\& \quad (1+u(j))^2F_AF_B\nonumber \\ {{\mathbf {E}}}(O_s)&=\frac{1}{4}\Pi _{j=1}^{s-1}(1+u(j))F_AF_B \end{aligned}$$

are the expected number of ortholog pairs observed after the $n_A-1-s$ WGD in species *A* by which time there will have been $n_B-1-s$ WGD in species *B*. The coefficient 1/4 is specific to WGD;  other events require a different constant.

The three key factors in our improved model, terms in Eqs. (25) and (26), are $(1+u^A(s)),(1+u^B(s))$ and $(1+u(s-1)$. Between the two successive WGD, at generation $s-1$ in the pre-speciation genome, and $s+1$ in genome A and also $s+1$ in genome B—though the two events are generally not synchronous, the same fractionation regime, in terms of rates, should hold, despite the speciation at generation *s*. Calculation of rates requires not only the *u*, but also a time $t_i$ associated with each event *i*. Writing

27 $$\begin{aligned} -\log u(s-1) & = \rho (t_s-t_{s-1}),\nonumber \\ -\log u^A(s) & = \rho _A(t^A_{s+1} -t_s),\nonumber \\ -\log u^B(s) & = \rho _B(t^B_{s+1} -t_s), \end{aligned}$$

our model presumes $\rho =\rho _A=\rho _B$. The same proportional rate should hold before and after speciation, since speciation is a population-level event in the first instance, not involving any genome-level changes, in contrast with WGD.

### The distribution of similarities

The goal of this work is to understand fractionation, so that if at the time of observation we could count the *i*- pairs for $i\ge 1$, we could use Eqs. like (19–23) as a basis for making inferences about the $u_j^{(i)}$. But although we can observe all the paralogous pairs, as well as the orthologous pairs if two species are involved, we cannot *directly* observe at which WGD or speciation event each pair originated. Instead, what we observe at generation *n* (or $n+1$ in the case of orthology, or later if there have been WGD in the daughter species) is a measure *p* of similarity (e.g., the proportion of identical nucleotides in the aligned coding sequences) between each pair of genes in the population. Because of how sequence similarity decays by random substitutions of nucleotides, we can expect an approximately exponential decline in *p* with time.

Thus if the distribution of gene pair similarities clusters around values $p_1<p_2<\cdots <p_{n-1}$, we can infer that these correspond to WGD events at some time $t_1<t_2<\cdots <t_{n-1}$ at generations $1,\ldots ,n-1$ of the branching process. And assuming a large sample of gene pairs, each of these clusters can be modelled by a normal distribution. The distribution of gene pairs is thus a mixture of $n-1$ normals.

Previous work assumed that the variance of the similarity of a gene pair was proportional to $p(1-p)$, but this did not provide a very good fit in practice. In the present paper, we do not assume any such relationship. Indeed, our strategy will be to identify the $t_i$ by a combination of techniques described in the next paragraph, and fix these in a standard maximum likelihood estimate of the variance and amplitude of each component of the mixture. This enables us to calculate the proportion of all the gene pairs in each component. We use these proportions, or frequencies derived by multiplying by the total number of pairs, as the numbers of *i* pairs, from which we can estimate the survival proportions using Eqs. (19–23).

### The mode as an estimator of $t_i$

The $t_i, i=1,\dots ,n-1$ are not inherent parts of the branching process model. But they are of course very important for the study of evolution and the estimation of rates.

There are well-established methods for decomposing a mixture of normals (or other predetermined distributions) into their component distributions [6]. Experience shows, however, that these methods, despite their built-in validation criteria, are not robust against non-normality, especially with genomic data, and tend to deliver spurious extra components, and components located in unlikely places. We will nevertheless make use of these methods, but in a way constrained to give appropriate results.

We will compare several genomes to each other. Our strategy is first to locate the $t_i$ in each comparison by picking out local modes in the distribution of similarities, guided by the knowledge that some of these $t_i$ are shared among several genome comparisons, since they reflect the same events. Then for each comparison, some of these estimates are refined by maximum likelihood methods, which also produce the amplitude and variance of the component. From these we can directly estimate how many gene pairs are 1-pairs, 2-pairs, etc. These numbers can then be used to produce estimates of the $u_j^{(i)}.$

Why use the mode? Because of overlapping tails, reminiscent of the mixing of generations, i.e., the decay of synchrony, in initially synchronized population, studied in the antediluvian literature [7], the means of the component distributions cannot be estimated by averaging, but can be identified as local modes in the overall distribution of gene pair similarities.

Estimating the local modes of an underlying distribution by using the modes of the sample involves a trade-off between precision and a proliferation of misleading modes. With gene pair similarities grouped into large bins, or averaged among moving windows of large size, the empirical distribution will be relatively smooth, and bonafide modes will be easily noticed. But a large bin size only indicates that the mode is somewhere in a large interval. With small bin sizes, or sliding window sizes, the position of the nodes are more precisely determined, but more subject to a proliferation of spurious nodes due to statistical fluctuation. Again, we control this problem by considering several related comparisons at a time.

## Results

### The evolution of the family Solanaceae

**Fig. 2:**
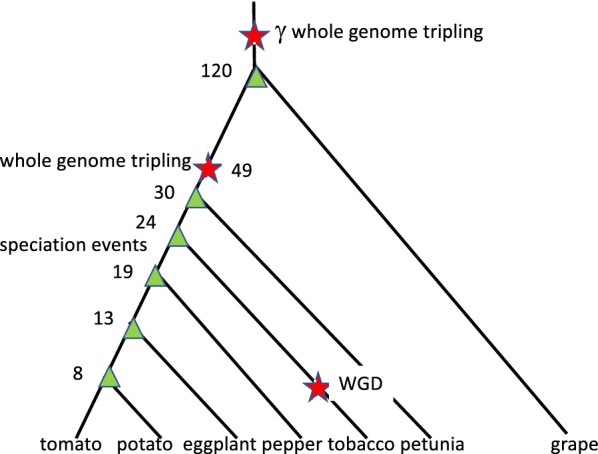
Phylogenetic relationships among the Solanaceae, showing WGD and speciation events. Numbers indicate millions of years from the event to the present, drawn from Figure 3 in [9], except for the interpolated age of eggplant speciation

The Solanaceae is a family of plants in the asterid order Solanales. This family is distinguished biologically by its early whole genome tripling, as indicated in Fig. 2, and scientifically by the fact that many of its species boast sequenced genomes, namely all the economically most important ones (cf [8]).

### The genomes

We use the SynMap software on CoGe, and thus have direct access to most of the data, in an appropriate format, among those available on the CoGe platform. Those genome data gathered elsewhere (cited below) were uploaded to a temporary private account on CoGe for purposes of the present research.

The tomato (*Solanum lycopersicum*) genome sequence and annotation [10] are considered the gold standard among the asterid genome projects. Although there is a recent update to version 3, we used the more familiar (from previous work) version 2.40.

The potato (*Solanum tuberosum*) genome [11] is also a high quality sequence has now been fully assembled into pseudomolecules (version 4.03).

The tobacco (*Nicotiana benthamiana*) genome was sequenced some years ago [12], but its sequence and annotation have been updated and made available for comparative purposes, together with the petunia (*Petunia hybrida* genome [9], both via SGN—the Sol Genomics Network https://solgenomics.net. Among the Solanaceae genomes studied here, only tobacco has undergone a WGD since the original Solanaceae tripling.

The pepper genome (*Capiscum annuum* version 1.55) [13] is drawn from a genus closely related to *Solanum*. We had no access to any updated version of this, and the quality of assembly and annotation is not as complete as those listed above.

A draft version of the eggplant genome (*Solanum melongena*) has also been available for some time [14], and this is what we use here despite its quality not measuring up to more recent standards, although a new version is available for browsing via SGN, with restrictions against comparative use awaiting the writing up and publication of the project.

As an outgroup, we use the grapevine (*Vitis vinifera*) genome [15], one of the first flowering plant genomes to be sequenced (in 2007), and one that has proven to be extraordinarily conservative, both with respect to mutational rate and to rearrangement of chromosomal structure. Indeed, the structure of the 19 grape chromosomes resembles in large measure that of the 21 chromosomes of the ancestor of the core eudicots, resulting from a tripling of a seven-chromosome precursor [16]. This is known as the “$\gamma$” tripling. Over half of the known flowering plants, including the Solanaceae, belong to this group.

### The comparisons

We applied SynMap to all pairs of the seven genomes and also compared each genome with itself (with the exception of eggplant, because of technical difficulties). We used the default parameters, which are fairly strict in ensuring that all pairs were part of a syntenic block, and thus created at the same time. This excluded duplicate gene pairs that may have been created individually, at some time other than during a WGD event.

**Fig. 3:**
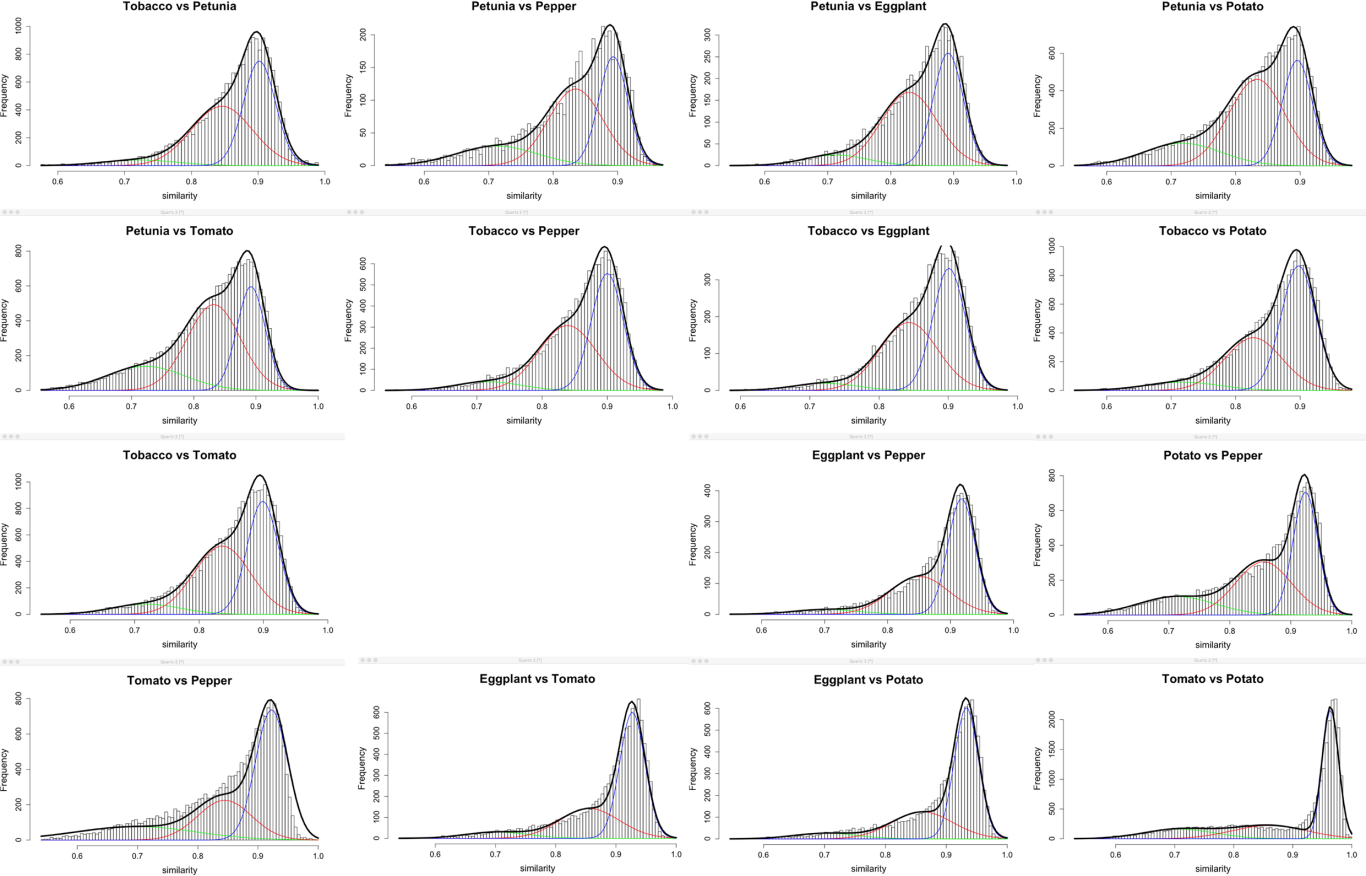
Distribution of ortholog similarities in comparisons among six Solanaceae genomes, with normal distributions fitted to similarities generated by each WGD and speciation event

**Fig. 4:**
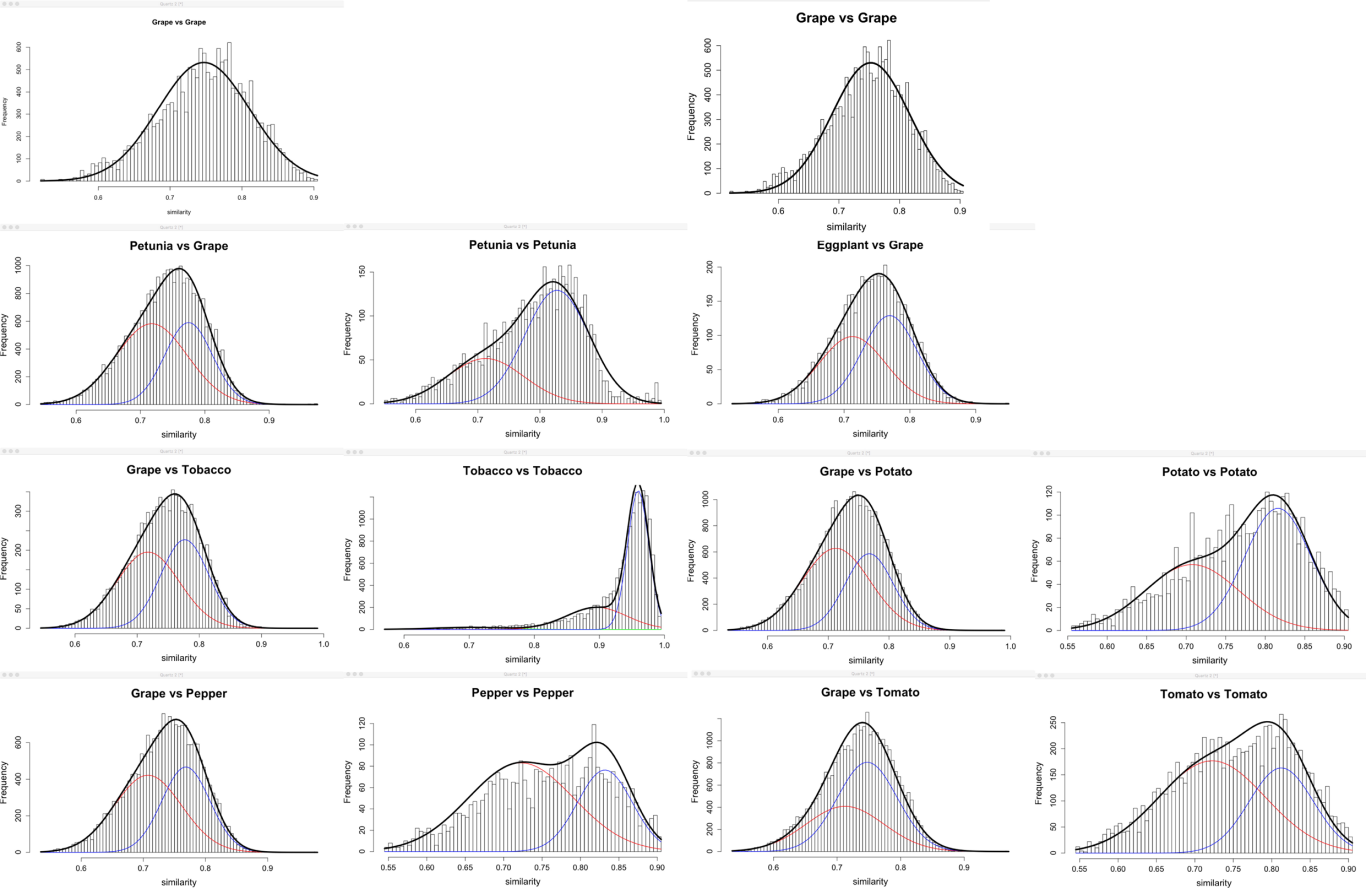
Distribution of paralog similarities in five$^*$ Solanaceae genomes and in grape, with normal distributions fitted to similarities generated by each WGD. This is compared to ortholog similarities in each Solanaceae genome versus grape. Two grape panels represent two slightly different fits to the data. Note the Y-axis in the tobacco self-comparison is out of proportion with the rest, because of its recent WGD. $^*$We were unable to run SynMap for eggplant self-comparison

The results are shown in Figs. 3 and 4. In Fig. 3, we note the relative stability of the $\gamma$ and Solanaceae tripling-based distributions, but the narrowing of the speciation-based distributions as speciation time approaches the present.

In Fig. 4, we note the conservatism of grape, which retains higher similarities for $\gamma$ paralogs than the Solanaceae. That the $\gamma$-based orthologs in the Solanaceae comparisons with grape all suggest equally remote speciation times, rather than manifesting a compromise with the more recent grape-versus-grape values indicates that the Solanaceae ancestor underwent a period of relatively rapid evolution.

**Fig. 5:**
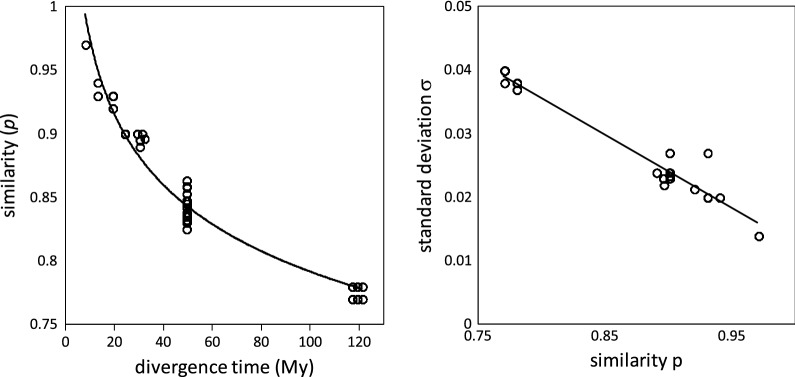
Left: Similarity of orthologs as a function of speciation time. Divergence times taken from Figure 3 in [9]. Right: Relation of standard deviation to component mean

We compiled the characteristics—$p, \sigma ,$ number (and overall proportion) of pairs—for each component in each of the analyses in Figs. 3 and 4. Of those in Fig. 3, only the results for the speciation (most recent) event are displayed in Table 1. Figure 5 shows the relation between *p* and divergence time for the speciation event pertinent to each pair of genomes, and their common earlier WGD.

**Table 1:**
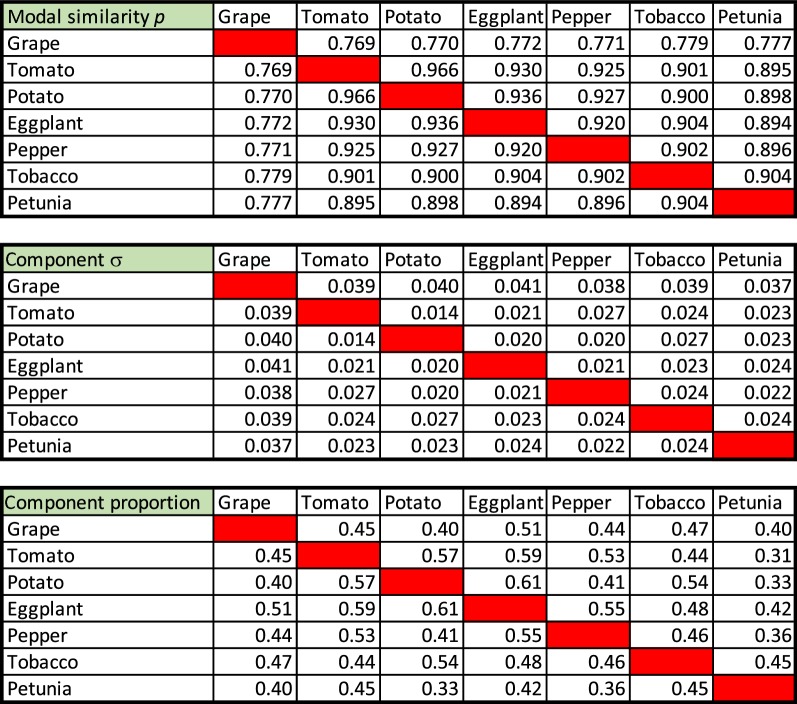
Characteristics inferred for speciation event distributions

On the left of Fig. 5, the cluster of points around 120 My represents the gene pairs generated by the $\gamma$ tripling event pre-dating all core eudicots, too remote in time to be distinguished from the speciation of the ancestor of grape and the ancestor of the Solanaceae. Points near the centre represent the Solanaceae tripling. Scattered points at more recent times indicate the speciation events among the six Solanaceae species.

The trend line in the figure is $p=1.2{\mathrm{e}}^{-0.09t}$, which fits well, although the coefficient of the exponential is greater than expected (i.e., 1.0). The right of Fig. 5 suggests that the standard deviation of the component normals are linearly related to their modes (and hence their means). The speciation data for modal values unequivocally support the phylogeny in Fig. 2, e.g., as calculated by neighbour joining (not shown).

### Fractionation rates

We calculated maximum likelihood estimates for $u_2^{(1)}, u_2^{(2)}$ and $u_2^{(3)}$, based on component proportions like those in the bottom section of Table 1. Because there are only two independent proportions per comparison, pertaining to $t_1, t_2$ and $t_3$, and an estimate of the number of unpaired genes (predicted by the model in Eq. 18), we could not also infer the $u_3^{(i)}$, and simply assumed $u_3^{(1)}=\left(u_2^{(1)}\right)^2$ and $u_3^{(2)}=\left(u_2^{(2)}\right)^2$, on the premise that the small probability of two additional progeny surviving (beyond the one essential to avoid extinction) would be approximately the product of their individual probabilities.

**Table 2:**
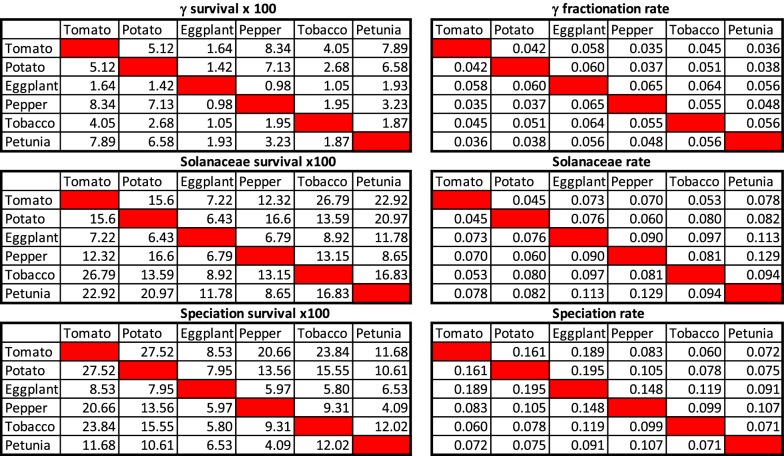
Estimates of survival (left) and of fractionation rates (right)

These event-specific and species-specific survival parameters $u_j^{(i)}$ on the left of Table 2 are directly estimable from the distribution statistics, and reveal much about the difference between the event and the species pairs, but our ultimate interest is in fractionation rates, which we denote $\rho$, and their consistency or variability. In general,

28 $$\begin{aligned} u(t) & = {\mathrm{e}}^{-\rho t}\nonumber \\ \rho & = \frac{-\ln u(t)}{t}. \end{aligned}$$

When we apply this rule to the survival rates in the table, using the time intervals derived from [9], we derive the fractionation rates on the right of the table. From the sections of Table 2 on survival we observe:

- The 15 estimates of survival between $\gamma$ and the Solanaceae tripling are systematically much lower than the survival between the latter tripling and speciation, and after speciation.
- The early survival figures are quite variable; a major cause of this is the quality of the genome sequencing, assembly and annotation, so that comparisons of the draft genome sequence of eggplant, for example, apparently miss many of the gene pairs generated by $\gamma$.
- The high rates of survival in the comparisons involving petunia or tobacco over the time interval between the Solanaceae tripling and speciation clearly reflect the shorter time interval before their respective speciation events.
- The speciation survival results reflect, as expected, phylogenetic relationships, though imperfectly, due in part to sequence and annotation quality, and in part due to the amplification of the number of pairs in the recent tobacco WGD.

From the sections of Table 2 on fractionation rates we observe:

- A large reduction of variability (compared to survival) in the results for the inter-tripling interval, due only to the logarithmic transform.
- A large, but not complete, reduction in the difference between the two periods of fractionation, due to the normalization by the time span. This is compatible with the idea that fractionation rates may be universally constrained to a relatively narrow range of values.
- The high rates of post-speciation ortholog loss within *Solanum*, and the relatively low rates for the comparisons involving petunia or tobacco, suggest that the process initially proceeds more quickly than fractionation, or levels off after a certain point, or both.

**Fig. 6:**
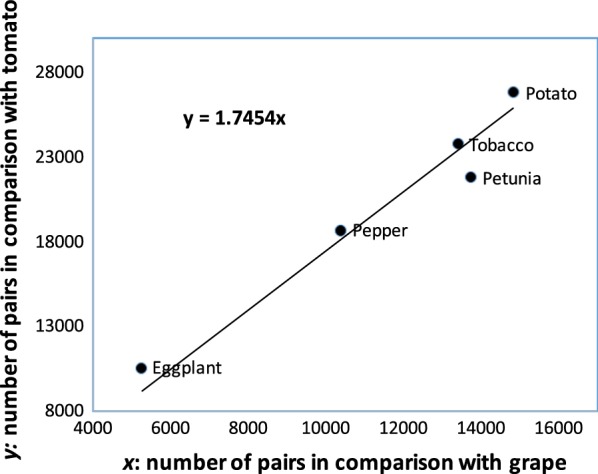
Estimating amplification factor due to *Solanum* triplication, by plotting number of gene pairs in comparisons with tomato against comparisons with grape

The modeling leading to Eq. (26) suggests that if only one of species A or B, undergoes another post-speciation WGD, we should be able to estimate the amplifying factor. Figure 6 suggests that tomato, which has undergone a WGT since its ancestral speciation from grape, has $F_A=1.75$. This results is confirmed if we substitute potato instead of tomato, but the great variability in genome quality precludes any meaningful results in other comparisons. In particular, we could not detect an effect of the recent tobacco WGD. Thus this kind of analysis must await the availability of a collection of related genomes with comparably high quality genome sequence.

## Conclusions

We modelled the process of fractionation to account for the distribution of similarities between paralog or ortholog gene pairs after a number of whole genome doublings, triplings, etc., each followed by a period of duplicate gene loss. The model is a discrete-time branching process, with its synchronous reproduction events across the population. Fractionation over the inter-generation interval is accounted for by the probability distribution on the number of offspring, interpreted instead as survival probabilities applied to a fixed number of offspring.

The observations of gene pair similarities consist of a mixture of normals, each component generated by one event, with the event time estimated by the sequence divergence from the event to the present. Despite the overlapping distributions, we can estimate the mean (via a local mode), standard deviation and proportion of the sample.

We then use these parameters to estimate survival probabilities for gene pairs from one event to the next. From the survival data we can then estimate fractionation rates, the number of gene pairs lost per unit time.

We apply our ideas to six genomes from the family Solanaceae and outlier grape. The SynMap program on the CoGe platform produces the distribution of similarities of syntenically validated paralogs and orthologs to feed into our analysis. The 21 pairwise genome comparisons produce a highly consistent picture of the creation and loss of duplicate gene pairs. The survival probabilities and fractionation rates are eminently interpretable in terms of phylogenetic considerations. This work has now been replicated for the family Malvaceae [17].

Based on our methods and results, we can accurately characterize fractionation rates, something first attempted some years ago [18]. Indeed, we are now in a position to question to what extent fractionation embodies clocklike behaviour.

## Data Availability

The datasets generated during and/or analysed during the current study are available in the CoGe repository, https://genomevolution.org/coge/.
